# Distributed Nonconvex Optimization for Control of Water Networks with Time-coupling Constraints

**DOI:** 10.1007/s11269-024-03985-8

**Published:** 2024-09-23

**Authors:** Bradley Jenks, Aly-Joy Ulusoy, Filippo Pecci, Ivan Stoianov

**Affiliations:** 1 Civil and Environmental Engineering, Imperial College London, London, SW7 2BU UK; 2 Andlinger Center for Energy and the Environment, Princeton University, Princeton, NJ 08544 USA

**Keywords:** Distributed optimization, Nonconvex optimization, Alternating direction method of multipliers, Water distribution networks

## Abstract

In this paper, we present a new control model for optimizing pressure and water quality operations in water distribution networks. Our formulation imposes a set of time-coupling constraints to manage temporal pressure variations, which are exacerbated by the transition between pressure and water quality controls. The resulting optimization problem is a nonconvex, nonlinear program with nonseparable structure across time steps. This problem proves challenging for state-of-the-art nonlinear solvers, often precluding their direct use for near real-time control in large-scale networks. To overcome this computational burden, we investigate a distributed optimization approach based on the alternating direction method of multipliers (ADMM). In particular, we implement and evaluate two algorithms: a standard ADMM scheme and a two-level variant that provides theoretical convergence guarantees for our nonconvex problem. We use a benchmarking water network and a large-scale operational network in the UK for our numerical experiments. The results demonstrate good convergence behavior across all problem instances for the two-level algorithm, whereas the standard ADMM approach struggles to converge in some instances. With an appropriately tuned penalty parameter, however, both distributed algorithms yield good quality solutions and computational times compatible with near real-time (e.g. hourly) control requirements for large-scale water networks.

## Introduction

Water distribution networks (WDNs) are undergoing a shift towards dynamic control, enabling them to adapt to various operational objectives in near real-time (Giudicianni et al. 2020; Bui et al. 2022; Ulusoy et al. 2023). This shift is essential to maintain optimal operations amid the increasing challenges posed by asset deterioration, regulatory compliance, and constrained resources. A critical concern for water companies is the management of discolouration risk, which can significantly impact water quality and customer satisfaction (Boxall et al. 2023). This concern has motivated a growing body of research on the design and control of self-cleaning networks for mitigating discolouration risk (Abraham et al. 2016, 2018; Jenks et al. 2023a). Recently, a multi-objective optimization problem investigated the integration of self-cleaning operations with pressure management, a widely adopted practice for reducing leakage in WDNs (Jenks et al. 2023b). A major challenge identified was the considerable fluctuations in operating pressures that could arise due to conflicting hydraulic states associated with pressure and self-cleaning controls. These dynamics raise concerns for water companies, as they must weigh the benefits of dynamic control against the reliability challenges of aging infrastructure.

There is growing evidence that suggests mean operating pressure and pressure variation influence pipe break rates. For example, Rezaei et al. (2015) found a positive correlation between pressure variation (up to $25 \, {\text {m}}$) and breaks caused by longitudinal cracks in operational WDNs in the UK. Similarly, Martínez-Codina et al. (2016) identified pressure range as the most statistically significant factor in pipe breaks. Jara-Arriagada and Stoianov (2021) demonstrated a $61\%$ reduction in cast iron pipe breaks for a $10 \, {\text {m}}$ decrease in pressure range, highlighting the benefits of active pressure management. These findings are relevant to our work, as changes in hydraulic states resulting from pressure and self-cleaning controls have been shown to significantly affect operating pressures (Wright et al. 2015; Abraham et al. 2016; Jenks et al. 2023b). Given its role in infrastructure reliability, pressure variation has also been considered as an objective in the design of dynamically adaptive WDNs (Pecci et al. 2017).

Introducing pressure variation constraints in the control problem couples otherwise independent time steps. The resulting time-coupled, nonconvex optimization problem often precludes the direct application of state-of-the-art nonlinear solvers. Specifically, these solvers become incompatible with near real-time control implementations in large-scale networks which require fast and scalable solution methods. In the literature, similar optimization problems have been addressed in the context of pump scheduling where tank levels are coupled by flow at consecutive time steps. For example, Zessler and Shamir (1989) and Nitivattananon et al. (1996) applied temporal decomposition and an iterative optimality technique to solve the time-coupled pump scheduling problem. In Ghaddar et al. (2015), a Lagrange decomposition method was integrated with a simulation-based feasibility search. Similarly, the alternating direction method of multipliers (ADMM) was introduced in Fooladivanda and Taylor (2018) to decouple feasibility constraints from the objective of minimizing pumping energy costs. ADMM was also applied in Zamzam et al. (2019) to decompose a coupled water-power network flow problem.

ADMM is a well-known decomposition-coordination technique for solving large-scale convex optimization problems using distributed computing architectures (Boyd et al. 2010). Our problem is particularly suitable for ADMM since it exhibits a block structure that can be decomposed into smaller subproblems. These subproblems are efficiently distributed among different computing agents, while a coordination step ensures information consensus at each iteration. However, the presence of nonconvexity in many engineering applications, including the operation of water networks, may prevent a standard ADMM implementation from converging to a stationary solution (Wang et al. 2019). Recently, the literature has explored theoretical convergence properties of ADMM for different classes of nonconvex problems. The majority of these studies, however, concern problems where nonconvexity resides solely in the objective function (Li and Pong 2015; Hong et al. 2016; Themelis and Patrinos 2020; Wang et al. 2019). For nonconvex constraints, ADMM convergence has only been established under relatively strong assumptions (Magnusson et al. 2016), which are often difficult to prove in practice. A promising approach to overcome these convergence issues, while preserving the desirable properties of ADMM, is the two-level distributed algorithm proposed in Sun and Sun (2023). This ADMM variant introduces slack variables in the consensus constraint to form a structure compliant with known ADMM convergence properties. An outer level augmented Lagrangian method (ALM) then drives the slack variables to zero using an amplifying penalty scheme. Recent work has successfully applied this two-level ADMM variant to optimal power flow problems (Sun and Sun 2021; Gholami et al. 2023) and large-scale model predictive control (MPC) implementations (Tang and Daoutidis 2022).

This paper presents a new control model for optimizing pressure and water quality operations in dynamically adaptive WDNs. Our formulation imposes a set of time-coupling constraints to manage pressure variations caused by the transition between pressure and water quality control objectives. We then investigate a distributed optimization approach to solve the resulting time-coupled, nonconvex problem, as state-of-the-art nonlinear solvers struggle to find feasible solutions. In particular, we implement a standard ADMM scheme and a two-level variant that provides convergence guarantees for our nonconvex problem. We demonstrate the computational performance of the distributed algorithms using a benchmarking water network and a large-scale operational network in the UK. Our analysis explores the empirical convergence behaviour of different ADMM penalty parameters, highlighting the robustness of the two-level algorithm to varying algorithmic parameters. With an appropriately tuned penalty parameter, however, both distributed algorithms exhibit superior computational performance compared to a centralized solution approach using a state-of-the-art non-linear optimization solver. We conclude discussing the application of distributed optimization for near real-time control in large-scale water networks.

The rest of the paper is organized as follows. Section 2 introduces the problem formulation, including the network model, operational objectives, and time-coupling constraints. In Section 3, we describe the standard ADMM and two-level distributed algorithms implemented to solve the time-coupled, nonconvex problem. In Section 4, we present case study data and the computational setup of numerical experiments. Lastly, in Section 5, we evaluate the performance of the distributed algorithms and discuss considerations for their practical implementation.

## Problem Formulation

In the following, we formulate an optimization problem for scheduling pressure and self-cleaning controls in water networks.

### Water Network Model

We model a water network as a directed graph $\mathcal {G}(\mathcal {N}, \mathcal {P})$. The set $\mathcal {N}$ comprises $n_n$ junction and $n_0$ source nodes and the set $\mathcal {P}$ comprises $n_p$ pipe or valve links. Network connectivity is represented through link-node incidence matrices $A_{12} \in \mathbb {R}^{n_p \times n_n}$ and $A_{10} \in \mathbb {R}^{n_p \times n_0}$ for junction and source nodes, respectively. We define $A_{12}$ (and $A_{10}$) using the following convention

1 $$\begin{aligned} \begin{aligned}&A_{12}(j,i) = {\left\{ \begin{array}{ll} 1 & \text {if link }j \textrm{enters node }i\\ 0 & \text {if link }j \textrm{is not connected to node }i \\ -1 & \text {if link }j\textrm{leaves node }i. \end{array}\right. } \end{aligned} \end{aligned}$$

We consider a steady-state hydraulic model to simulate hydraulic states. Let ${\mathcal {T} = \{1,\dots , n_t\}}$ denote a finite control horizon, which represents a collection of discrete time steps spanning a moving window of size $n_t$. For each time step $t \in \mathcal {T}$, the vectors of link flows ${q_t \in \mathbb {R}^{n_p}}$ and hydraulic heads ${h_t \in \mathbb {R}^{n_n}}$ denote unknown hydraulic states. Known hydraulic conditions are given by vectors of nodal demands ${d_t \in \mathbb {R}^{n_n}}$ and source hydraulic heads ${h_{0t} \in \mathbb {R}^{n_0}}$. Moreover, we introduce control via (i) pressure control valves (PCVs), which modulate pressure in a single flow direction, and (ii) automatic flushing valves (AFVs), which discard water at designated locations. The vectors ${\eta _t \in \mathbb {R}^{n_v}}$ and ${\alpha _t \in \mathbb {R}^{n_f}}$ model local losses across $n_v$ PCV links and operational demands at $n_f$ AFV nodes, respectively. Matrices ${A_{13} \in \mathbb {R}^{n_p \times n_v}}$ and ${A_{14} \in \mathbb {R}^{n_n \times n_f}}$ map known actuator locations.

Hydraulic states $q_t$ and $h_t$ are governed by the following energy Eq. 2a and mass Eq. 2b conservation equations at time *t*:

2a $$\begin{aligned}&A_{12}h_t + A_{10}h_{0t} + \phi (q_t) + A_{13}\eta _t = 0, \end{aligned}$$

2b $$\begin{aligned}&A_{12}^T q_t - d_t - A_{14}\alpha _t = 0, \end{aligned}$$

where $\phi (\cdot )$ is a nonlinear function modelling frictional head loss across each link $j \in \mathcal {P}$, defined as

3 $$\begin{aligned} \phi _j(q_{j,t}) = r_j|q_{j,t}|^{n_j-1}q_{j,t}. \end{aligned}$$

For valve links, $n_j = 2$ and

4 $$\begin{aligned} r_j = \frac{8K_j}{g\pi ^2D_j^4}, \end{aligned}$$

where $K_j$ and $D_j$ denote the valve loss coefficient and diameter, respectively at link *j*. For pipe links, we apply the empirical Hazen-Williams formula, which has parameters $n_j = 1.852$ and

5 $$\begin{aligned} r_j = \frac{10.67L_j}{C_j^{1.852}D_j^{4.871}}, \end{aligned}$$

where $L_j$ is the length of link *j*; $C_j$ is the dimensionless H-W coefficient; and $D_j$ is the diameter of link *j*. Note that the semi-empirical Darcy-Weisbach formula can also be used to compute head loss across pipe links with no change to the problem formulation.

We introduce upper and lower bounds on continuous variables $h_t$, $\eta _t$, and $\alpha _t$ to define the feasible solution space for all $t \in \mathcal {T}$,

6a $$\begin{aligned}&h^{\min }_t \le h_t \le h^{\max }_t, \end{aligned}$$

6b $$\begin{aligned}&\eta ^L_t \le \eta _t \le \eta ^U_t, \end{aligned}$$

6c $$\begin{aligned}&0 \le \alpha _t \le \alpha ^{\max }_t, \end{aligned}$$

where $h^{\min }_t$ is set to the minimum regulatory pressure and ${h^{\max }_t = \max _{i = 1, \dots , n_0}(h_{0t})_i \times \mathbbm {1}_{n_n}}$; $\eta ^L_t$ and $\eta ^U_t$ are established from corresponding $h^{\min }_t$ and $h^{\max }_t$ values at the upstream and downstream nodes of PCV links; and $\alpha ^{\max }_t$ sets the maximum flushing rate at AFV nodes. Note that $h^{\min }_t$ and $\alpha ^{\max }_t$ are defined on the basis of local regulatory and network conditions. Additionally, we enforce control direction at PCV links by introducing the following bi-linear constraint on flow $q_t$ and local loss $\eta _t$ variables for all $t \in \mathcal {T}$:

7 $$\begin{aligned} q_{j,t}(A_{13}\eta _t)_j \ge 0, \quad \forall j \in \mathcal {P}. \end{aligned}$$

### Control Objectives

In this work, we focus on the coordination of two control objectives. The first objective concerns hydraulic pressure, which plays a critical role in background leakage and pipe burst frequency (Schwaller and van Zyl 2015). We define the network’s average zone pressure (AZP) as a surrogate measure of pressure-induced leakage and pipe circumferential stress (Wright et al. 2015), expressed as the linear function

8 $$\begin{aligned} f_{\text {AZP}}(h_t) := \sum _{i=1}^{n_n} w_{\text {AZP}i} (h_{i,t} - \zeta _i), \end{aligned}$$

where $h_{i,t}$ is the computed hydraulic head for node *i* at time step *t*; $\zeta _i$ is the elevation of node *i*; and $w_{\text {AZP}i}$ is a coefficient weighting node *i* by the length of its connected links (Wright et al. 2015, Equation 5).

The second objective aims to reduce discolouration risk via self-cleaning velocities. Recent research has investigated different optimization problems for maximizing the self-cleaning capacity (SCC) of a network (Abraham et al. 2016, 2018; Jenks et al. 2023a). The SCC objective function is defined as the length of pipe with flow velocities exceeding a threshold sufficient to mobilize accumulated material (Vreeburg et al. 2009; Blokker et al. 2010). In order to use gradient-based optimization methods, Abraham et al. (2016) proposed a smooth sum of logistic functions to approximate the SCC objective. This nonconvex approximation is written as

9 $$\begin{aligned} f_{\text {SCC}}(q_t) := \sum _{j=1}^{n_p}w_{\text {SCC}j} \left( \psi ^+_{j}\left( \frac{q_{j,t}}{s_j}\right) + \psi ^-_j\left( \frac{q_{j,t}}{s_j}\right) \right) , \end{aligned}$$

where $\psi ^+_{j}$ and $\psi ^-_{j}$ are logistic functions in the positive and negative flow directions, respectively, for link *j* (see Jenks et al. 2023a for details); $q_{j,t}$ is the flow conveyed by link *j* at time step *t*; $s_j$ is the cross-sectional area of link *j*; and $w_{\text {SCC}j}$ is a coefficient normalizing the length of link *j* to the entire network.

**Fig. 1:**
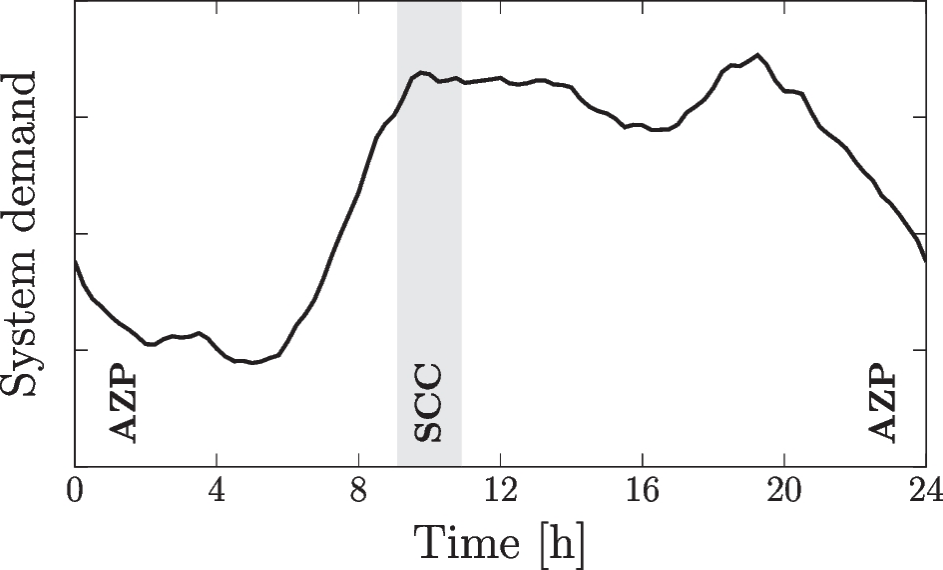
AZP (primary) and SCC (secondary) control modes across the 24-hour control horizon $\mathcal {T}$. Note that the SCC window (shaded area) can be modified to align with network specific conditions

Given that background leakage is a continuous phenomenon, we designate AZP as the primary (or continuous) control mode. Conversely, we set SCC as the secondary (or periodic) control mode, activated within a predefined 1-hour window. This coordination strategy is based on the assumption that achieving self-cleaning velocities daily (or every other day) mitigates the risk of discolouration in WDNs (Vreeburg et al. 2009; Blokker et al. 2010). Figure 1 illustrates the 24-hour control horizon, with system demand overlaid to highlight SCC activation during peak demand.

Here, we activate SCC controls between 09:30 to 10:30 to leverage the high flow velocities from peak demands. However, the duration and/or activation of this window can be easily modified in the problem formulation. Additionally, the SCC window may vary depending on the spatial distribution of demand characteristics in the network, particularly when targeting self-cleaning conditions for a specific subset of links.

### Time-coupling Constraints

We introduce a set of time-coupling constraints to manage nodal pressure range across the 24-hour control horizon. These constraints are defined by the following inequality:

10 $$\begin{aligned} \max _{t\in \mathcal {T}}(h_{i,t}) - \min _{t\in \mathcal {T}}(h_{i,t}) \le \delta , \quad \forall i \in \mathcal {N}, \end{aligned}$$

where $\delta$ corresponds to a specified pressure range tolerance. For ease of notation, we introduce the set $\bar{\mathcal {X}} = \{x \in \mathbb {R}^{n_t \times n_n} \; | \; \max _{t\in \mathcal {T}}(x_{i,t}) - \min _{t\in \mathcal {T}}(x_{i,t}) \le \delta , \; \forall i \in \mathcal {N}\}$. Note that, in our practical implementations, we reformulate Eq. 10 as a set of linear constraints by introducing auxiliary variables $c_{ui}$ and $c_{li}$ and setting

11a $$\begin{aligned}&h_{i,t} \le c_{ui}, \quad \forall t \in \mathcal {T} \end{aligned}$$

11b $$\begin{aligned}&h_{i,t} \ge c_{li}, \quad \forall t \in \mathcal {T} \end{aligned}$$

11c $$\begin{aligned}&c_{ui} - c_{li} \le \delta . \end{aligned}$$

The choice of pressure range tolerance $\delta$ is based on its observed impact on pipe breaks, as demonstrated in statistical analyses from previous studies (Martínez-Codina et al. 2016; Rezaei et al. 2015; Jara-Arriagada and Stoianov 2021). We explore the effect of $\delta$ on the resulting control actions (*u*) and hydraulic states (*q*, *h*) in Section 5. Additionally, we do not consider the frequency of pressure variation events since our problem focuses on changes in daily (steady-state) operating pressures. System dynamics were omitted on the assumption that control inputs in practice are designed to be gradual and thus mitigate the occurrence of sudden pressure changes.

### Control Problem

This work aims to optimize the AZP-SCC control schedule described in Section 2.2 while managing temporal pressure variations through time-coupling constraints described in Section 2.3. The corresponding optimization problem can be formulated in compact form as

12a $$\begin{aligned}&\text {minimize}&\quad&\sum _{t \in \mathcal {T}} f_t(q_t, h_t) \end{aligned}$$

12b $$\begin{aligned}&\text {subject to} & (q_t, h_t, u_t) \in \mathcal {X}_t, \quad \forall t \in \mathcal {T} \end{aligned}$$

12c $$\begin{aligned} & &h \in \bar{\mathcal {X}}, \end{aligned}$$

where $f_t:= -f_{\text {SCC}}$ if $t \in \mathcal {T}_{\text {SCC}}$ and $f_t:= f_{\text {AZP}}$ if $t \notin \mathcal {T}_{\text {SCC}}$; $u_t = (\eta _t, \alpha _t)$ defines the control variables at time *t*; $\mathcal {X}_t$ collects the hydraulic constraints described in Eqs. 2a, 2b, 6, and 7; and $\bar{\mathcal {X}}$ represents the set of time-coupling constraints in Eq. 10.

Problem Eq. 12 is a nonconvex, nonlinear programming (NLP) problem whose size grows with the number of discrete time steps in $\mathcal {T}$. A large number of time steps may be required to capture the diurnal periodicity of loading conditions as well as leverage the high-resolution frequencies of modern network sensing. In addition, time-coupling constraints Eq. 12c introduce a nonseparable structure. These characteristics make problem Eq. 12 challenging to solve for near real-time control implementations in large-scale water networks.

## Distributed Optimization

**Fig. 2:**
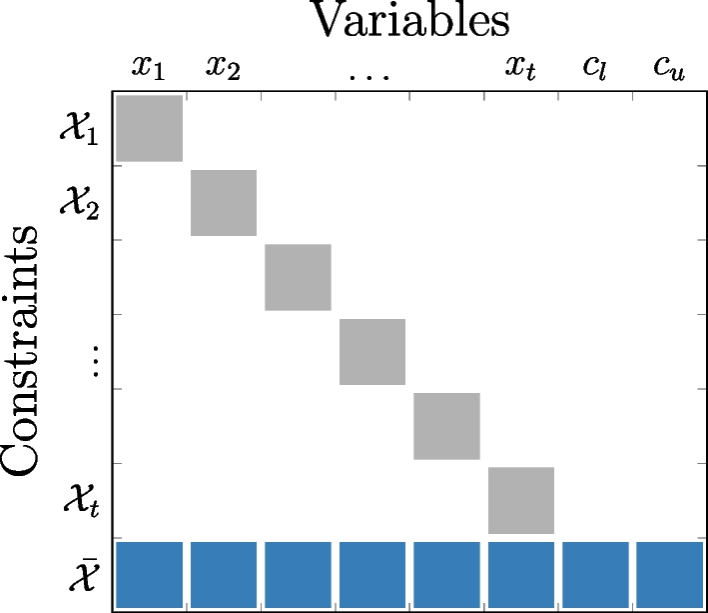
Block structure of problem Eq. 12 with time-coupling constraints $\bar{\mathcal {X}}$

Problem Eq. 12 exhibits a special structure in which the only coupling between time steps is present in constraints Eq. 12c. This nearly separable block structure, illustrated in Fig. 2, is well-suited for decomposition-coordination procedures. In this work, we apply the alternating direction of multipliers (ADMM) algorithm, a widely adopted decomposition-coordination procedure for handling problems with coupling constraints (Eckstein and Bertsekas 1992; Boyd et al. 2010).

We first describe a standard ADMM implementation by introducing a global variable copy (or variable splitting) in Section 3.1. This decomposes problem Eq. 12 into a series of smaller subproblems independent of time, enabling the use of distributed computing to enhance computational performance. Since the standard ADMM implementation does not provide theoretical convergence guarantees for nonconvex problems, we also implement the two-level ADMM variant (Sun and Sun 2021, 2023) in Section 3.2, which is designed to handle problems with nonconvex constraints.

The following notation is used in the subsequent sections. The identity matrix of size *n* is denoted by $I_n$. The inner product of $x, y \in \mathbb {R}^n$ is denoted by $x^Ty$ or $\langle x, y \rangle$. We use $\Vert x\Vert := \sqrt{\langle x, x \rangle }$ to denote the Euclidean norm of $x \in \mathbb {R}^n$. The indicator function of constraint set $\mathcal {X}$ is denoted by $\mathbb {I}_{\mathcal {X}}(x)$, with $\mathbb {I}_{\mathcal {X}}(x) = 0$ if $x \in \mathcal {X}$ and $+\infty$ if $x \notin \mathcal {X}$. The projection operator onto a closed set $\mathcal {X}$ is denoted as ${\text {Proj}}_{\mathcal {X}}(x)$. Lastly, we define $[n] = \{1, \dots , n\}$.

### Standard ADMM

We first present a distributed reformulation of problem Eq. 12 by introducing a global copy of hydraulic state variables $\bar{h} \in \mathbb {R}^{n_n \times n_t}$ and the consensus constraint

13 $$\begin{aligned} h - \bar{h} = 0, \end{aligned}$$

After duplicating variables, problem Eq. 12 is equivalently written as

14a $$\begin{aligned}&\text {minimize}&\quad&\sum _{t \in \mathcal {T}} f_t(q_t,h_t) \end{aligned}$$

14b $$\begin{aligned}&\text {subject to} & h - \bar{h} = 0, \end{aligned}$$

14c $$\begin{aligned} & &(q_t, h_t, u_t) \in \mathcal {X}_t, \quad \forall t \in \mathcal {T} \end{aligned}$$

14d $$\begin{aligned} & &\bar{h} \in \bar{\mathcal {X}}, \end{aligned}$$

which decomposes the problem across time steps as time-coupling constraints $\bar{\mathcal {X}}$ are now strictly a function of duplicated variables $\bar{h}$.

A standard ADMM implementation minimizes the augmented Lagrangian of problem Eq. 14 (Boyd et al. 2010), defined as

15 $$\begin{aligned} \begin{aligned} L(q,h,u,\bar{h},y) =&\sum _{t \in \mathcal {T}} \Big \{ f_t(q_t,h_t,u_t) + \mathbb {I}_{\mathcal {X}_t}(q_t,h_t,u_t) + \langle y_t, h_t - \bar{h}_t \rangle \\&+ \frac{\rho }{2} \Vert h_t - \bar{h}_t\Vert ^2 \Big \} + \mathbb {I}_{\bar{\mathcal {X}}}(\bar{h}) \\ =&\sum _{t \in \mathcal {T}} L_t(q_t,h_t,u_t,\bar{h}_t,y_t) + \mathbb {I}_{\bar{\mathcal {X}}}(\bar{h}), \end{aligned} \end{aligned}$$

where $\rho > 0$ is a fixed penalty parameter; $y_t \in \mathbb {R}^{n_n}$ is the vector of dual variables associated with consensus constraint Eq. 14b, with vectors $y = [\{y_t\}_{t \in \mathcal {T}}]^T$; and $\mathbb {I}_{\mathcal {X}_t}(\cdot )$ and $\mathbb {I}_{\bar{\mathcal {X}}}(\cdot )$ are the indicator functions of hydraulic feasibility set $\mathcal {X}_t$ and time-coupling constraint set $\bar{\mathcal {X}}$, respectively. Given initial values $(q^k,h^k,u^k,\bar{h}^k,y^k)$, ADMM minimizes Eq. 15 by performing a sequence of steps over *k* iterations, as follows:

16a $$\begin{aligned} q_t^{k+1},h_t^{k+1}, u_t^{k+1}&:= \underset{\begin{array}{c} q_t,h_t,u_t \end{array}}{\mathop {\mathrm {arg\,min}}\limits } \; L_t(q_t,h_t,u_t,\bar{h}_t^k,y_t^k), \,\; \forall t \in \mathcal {T} \end{aligned}$$

16b $$\begin{aligned} \bar{h}^{k+1}&:= \underset{\begin{array}{c} \bar{h} \end{array}}{\mathop {\mathrm {arg\,min}}\limits } \; L(q^{k+1},h^{k+1},u^{k+1},\bar{h},y^k) \end{aligned}$$

16c $$\begin{aligned} y^{k+1}&:= y^k + \rho (h^{k+1} - \bar{h}^{k+1}). \end{aligned}$$

The sequence of ADMM steps can be efficiently handled by state-of-the-art optimization solvers. With $\bar{h}^k$ and $y^k$ fixed, locally optimal state $(q_t^{k+1},h_t^{k+1})$ and control $u_t^{k+1}$ variables can be independently computed for each nonconvex NLP subproblem in Eq. 16a. The NLP subproblems can therefore be distributed across multiple processes to improve computational performance (see Section 4 for distributed computing implementation used in this work). In the subsequent step, $h^{k+1}$ is broadcast to the coordination problem in Eq. 16b, which is a relatively small convex quadratic problem enforcing time-coupling constraints $\bar{h} \in \bar{\mathcal {X}}$. Lastly, dual variables $y^{k+1}$ are updated in Eq. 16c, with the fixed penalty parameter $\rho$ as the step size.

We terminate ADMM if solution $(q^k,h^k,u^k,\bar{h}^k,y^k)$ at iteration *k* satisfies the following criteria:

17a $$\begin{aligned}&\Vert \rho (\bar{h}^{k+1} - \bar{h}^k)\Vert \le \epsilon _{\text {d}} \end{aligned}$$

17b $$\begin{aligned}&\Vert h^k - \bar{h}^k\Vert \le \epsilon _{\text {p}}, \end{aligned}$$

where Eqs. 17a and 17b denote the dual feasibility and primal residuals, respectively; and $\epsilon _{\text {d}}$ and $\epsilon _{\text {p}}$ are positive tolerances.

Recent literature has explored theoretical convergence properties for nonconvex ADMM problems (Hong et al. 2016; Magnusson et al. 2016; Wang et al. 2019; Jiang et al. 2019). However, Sun and Sun (2021, 2023) demonstrate that problem Eq. 14 fails to satisfy the sufficient conditions necessary for ensuring convergence to a stationary solution. Specifically, the local constraints on the last block update in Eq. 16b prevent the use of the unconstrained optimality condition to link primal and dual variables. Although these conditions guarantee convergence, they are not necessarily required for ADMM to reach a stationary solution. Thus, we empirically examine the convergence behavior of the standard ADMM scheme from a practical perspective in Section 5.

### Two-level Algorithm

Here, we implement the two-level ADMM variant proposed in Sun and Sun (2023). Unlike the standard approach, the two-level algorithm provides theoretical convergence guarantees to a stationary solution under mild assumptions for the nonconvex setting.

We consider the following reformulation of problem Eq. 14 with slack variables $z \in \mathbb {R}^{n_n \times n_t}$

18a $$\begin{aligned}&\text {minimize}&\quad&\sum _{t \in \mathcal {T}} f_t(q_t,h_t) \end{aligned}$$

18b $$\begin{aligned}&\text {subject to} & h - \bar{h} + z = 0 \end{aligned}$$

18c $$\begin{aligned} & &(q_t, h_t, u_t) \in \mathcal {X}_t, \quad \forall t \in \mathcal {T} \end{aligned}$$

18d $$\begin{aligned} & &\bar{h} \in \bar{\mathcal {X}} \end{aligned}$$

18e $$\begin{aligned} & &z = 0. \end{aligned}$$

In this reformulation, the consensus constraint Eq. 18b involves three variable blocks $(h, \bar{h}, z)$. To preserve ADMM convergence properties, Sun and Sun (2023) consider constraint $z=0$ separately through an augmented Lagrangian relaxation (ALR) of problem Eq. 18. The ALR at iteration *m* is formulated as

19a $$\begin{aligned}&\text {minimize}&\quad&\sum _{t \in \mathcal {T}} \Big \{ f_t(q_t,h_t) + \langle \lambda _t^m, z_t \rangle + \frac{\beta ^m}{2} \Vert z_t\Vert ^2 \Big \} \end{aligned}$$

19b $$\begin{aligned}&\text {subject to} & h - \bar{h} + z = 0 \end{aligned}$$

19c $$\begin{aligned} & &(q_t, h_t, u_t) \in \mathcal {X}_t, \quad \forall t \in \mathcal {T} \end{aligned}$$

19d $$\begin{aligned} & &\bar{h} \in \bar{\mathcal {X}}, \end{aligned}$$

where $\lambda _t^m \in \mathbb {R}^{n_n}$ is the vector of dual variables associated with constraint Eq. 18e at iteration *m*, with vectors $\lambda = [\{\lambda _t\}_{t \in \mathcal {T}}]^T$; and $\frac{\beta ^m}{2} \Vert z_t\Vert ^2$ is a quadratic penalty term with $\beta ^m >0$ at iteration *m* and time step *t*. Relaxing constraint $z=0$, we can solve problem Eq. 19 via a three-block ADMM scheme that now satisfies the unconstrained optimality condition of the last variable block *z*.

Given $\lambda ^m$ and $\beta ^m$ at the *m*-th outer level iteration, ADMM minimizes the augmented Lagrangian function associated with problem Eq. 19, defined as

20 $$\begin{aligned} \begin{aligned} \hat{L}^m(q,h,u,\bar{h},z,y) =&\sum _{t \in \mathcal {T}} \Big \{ f_t(q_t,h_t,u_t) + \mathbb {I}_{\mathcal {X}_t}(q_t,h_t,u_t) + \langle \lambda _t^m, z_t \rangle \\&+ \frac{\beta ^m}{2} \Vert z_t\Vert ^2 + \langle y_t, h_t - \bar{h}_t + z_t \rangle \\&+ \frac{\rho ^m}{2} \Vert h_t - \bar{h}_t + z_t\Vert ^2 \Big \} + \mathbb {I}_{\bar{\mathcal {X}}}(\bar{h}) \\ =&\sum _{t \in \mathcal {T}} \hat{L}^m_t(q_t,h_t,u_t,\bar{h}_t,z_t,y_t) + \mathbb {I}_{\bar{\mathcal {X}}}(\bar{h}), \end{aligned} \end{aligned}$$

where $y_t \in \mathbb {R}^{n_n}$ is the vector of dual variables associated with constraint Eq. 19b, with vectors $y = [\{y_t\}_{t \in \mathcal {T}}]^T$; and $\rho ^m>0$ is the ADMM penalty parameter at iteration *m* of the outer level problem. The three-block ADMM updates $(q^k,h^k,u^k,\bar{h}^k,z^k,y^k)$ in sequence over *k* inner level iterations, with $\lambda ^m$, $\beta ^m$, and $\rho ^m$ kept constant at each outer level iteration *m*:

21a $$\begin{aligned} q_t^{k+1},h_t^{k+1}, u_t^{k+1}&:= \underset{\begin{array}{c} q_t,h_t,u_t \end{array}}{\mathop {\mathrm {arg\,min}}\limits } \; \hat{L}_t(q_t,h_t,u_t,\bar{h}_t^k,z_t^k,y_t^k), \,\; \forall t \in \mathcal {T} \end{aligned}$$

21b $$\begin{aligned} \bar{h}^{k+1}&:= \underset{\begin{array}{c} \bar{h} \end{array}}{\mathop {\mathrm {arg\,min}}\limits } \; \hat{L}(q^{k+1},h^{k+1},u^{k+1},\bar{h},z^k,y^k) \end{aligned}$$

21c $$\begin{aligned} z^{k+1}&:= \underset{\begin{array}{c} z \end{array}}{\mathop {\mathrm {arg\,min}}\limits } \; \hat{L}(q^{k+1},h^{k+1},u^{k+1},\bar{h}^{k+1},z,y^k) \end{aligned}$$

21d $$\begin{aligned} y^{k+1}&:= y^k + \rho ^m(h^{k+1} - \bar{h}^{k+1} + z^{k+1}). \end{aligned}$$

Similar to the standard ADMM implementation in Eq. 16, hydraulic state $(q_t^{k+1},h_t^{k+1})$ and control $u_t^{k+1}$ variables are decoupled across time steps $t \in \mathcal {T}$ in Eq. 21a and can thus be updated in a distributed manner. Under mild assumptions, which we show problem Eq. 18 satisfy in Appendix A, Sun and Sun (2023) prove that the inner level ADMM described in Eq. 21 converges to an approximate stationary solution of problem Eq. 19 when $\rho ^m = 2\beta ^m$.

In practice, we terminate ADMM if solution $(q^k,h^k,u^k,\bar{h}^k,z^k,y^k)$ at inner level iteration *k* satisfies the following stopping criteria:

22a $$\begin{aligned}&\Vert \rho ^m (\bar{h}^{k-1} + z^{k-1} - \bar{h}^k - z^k)\Vert \le \epsilon _1 \end{aligned}$$

22b $$\begin{aligned}&\Vert \rho ^m (z^{k-1} - z^k)\Vert \le \epsilon _2 \end{aligned}$$

22c $$\begin{aligned}&\Vert h^k - \bar{h}^k + z^k\Vert \le \epsilon _3. \end{aligned}$$

where $\epsilon _{i \in [3]}$ are positive tolerances.

However, constraint Eq. 18e may not necessarily be satisfied after ADMM successfully terminates at the inner level. A classical augmented Lagrangian method is therefore adopted to drive slack variables *z* to zero by updating $\lambda ^{m+1}$, as follows:

23 $$\begin{aligned} \lambda ^{m+1} = {\text {Proj}}_{[\underline{\lambda }, \overline{\lambda }]}(\lambda ^m + \beta ^m z^m), \end{aligned}$$

in which the projection onto a predetermined hypercube $[\underline{\lambda }, \overline{\lambda }]$ is introduced to ensure boundedness of dual variables and thereby the augmented Lagrangian function $\hat{L}$. We also amplify the penalty parameter $\beta ^m$ if $z^m$ has not sufficiently decreased from the previous iteration $z^{m-1}$. The $\beta ^{m+1}$ update step is written as

24 $$\begin{aligned} \begin{aligned}&\beta ^{m+1} = {\left\{ \begin{array}{ll} \gamma \beta ^m, \quad & \Vert z^m\Vert > \omega \Vert z^{m-1}\Vert \\ \beta ^m, \quad & \Vert z^m\Vert \le \omega \Vert z^{m-1}\Vert \end{array}\right. } \end{aligned} \end{aligned}$$

where $\gamma >1$ and $\omega \in [0,1)$ are tunable parameters. We terminate the outer level ALM if solution $(q^m,h^m,u^m,\bar{h}^m,z^m)$ satisfies the primal residual stopping criterion

25 $$\begin{aligned} \Vert h^k - \bar{h}^k\Vert \le \epsilon _p. \end{aligned}$$

In summary, we solve problem Eq. 18 in two levels: the inner level applies a three-block ADMM to find an approximate stationary solution to problem Eq. 19, with iterates indexed by *k*; and the outer level drives slack variables *z* to zero using a classic ALM framework, with iterates indexed by *m*. This two-level algorithm is repeated until the outer level stopping criterion Eq. 25 is met. Pseudocode for the two-level algorithm is presented in Algorithm 1.

**Algorithm 1:**
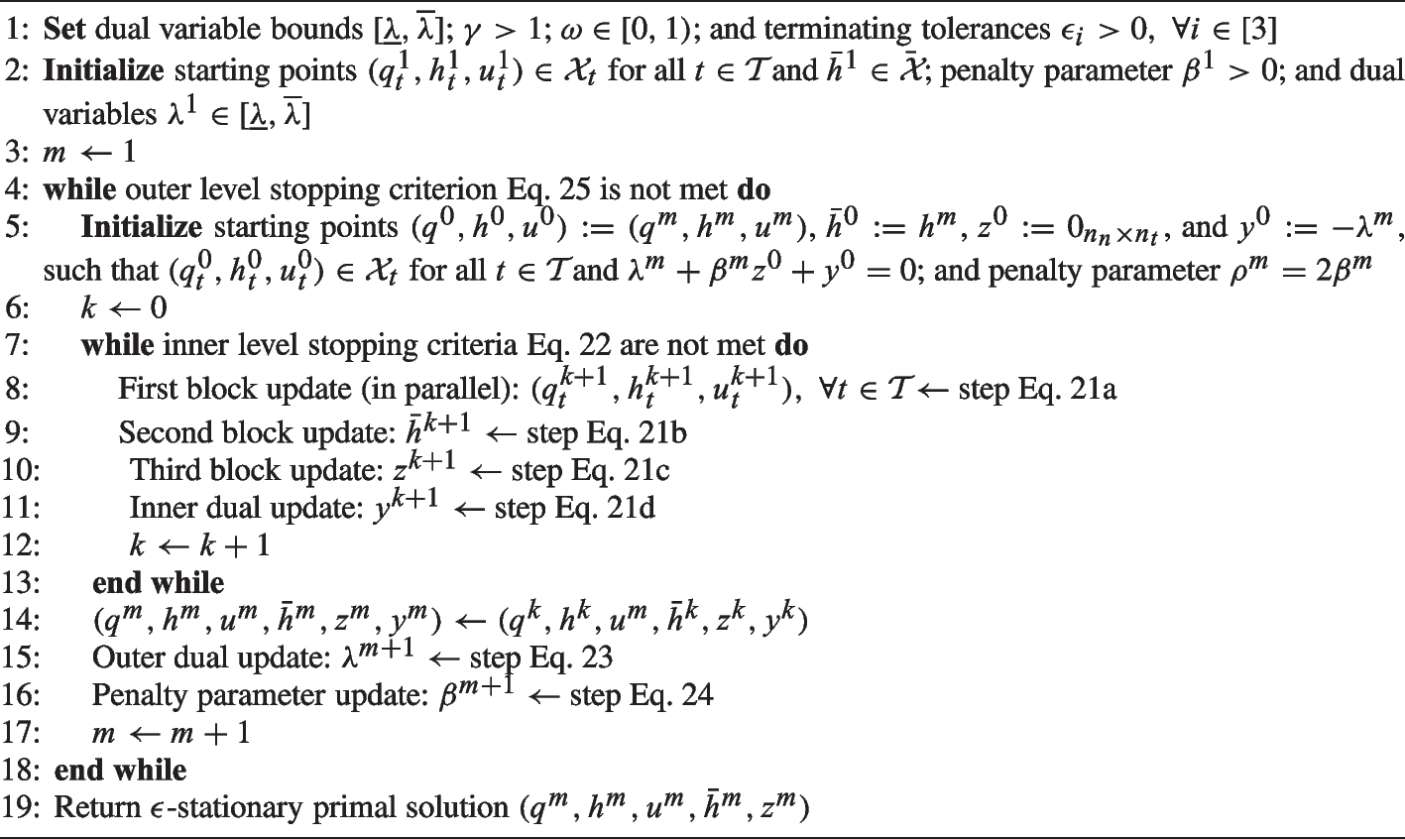
Two-level distributed algorithm.

## Numerical Experiments

### Case Studies and Problem Data

**Fig. 3:**
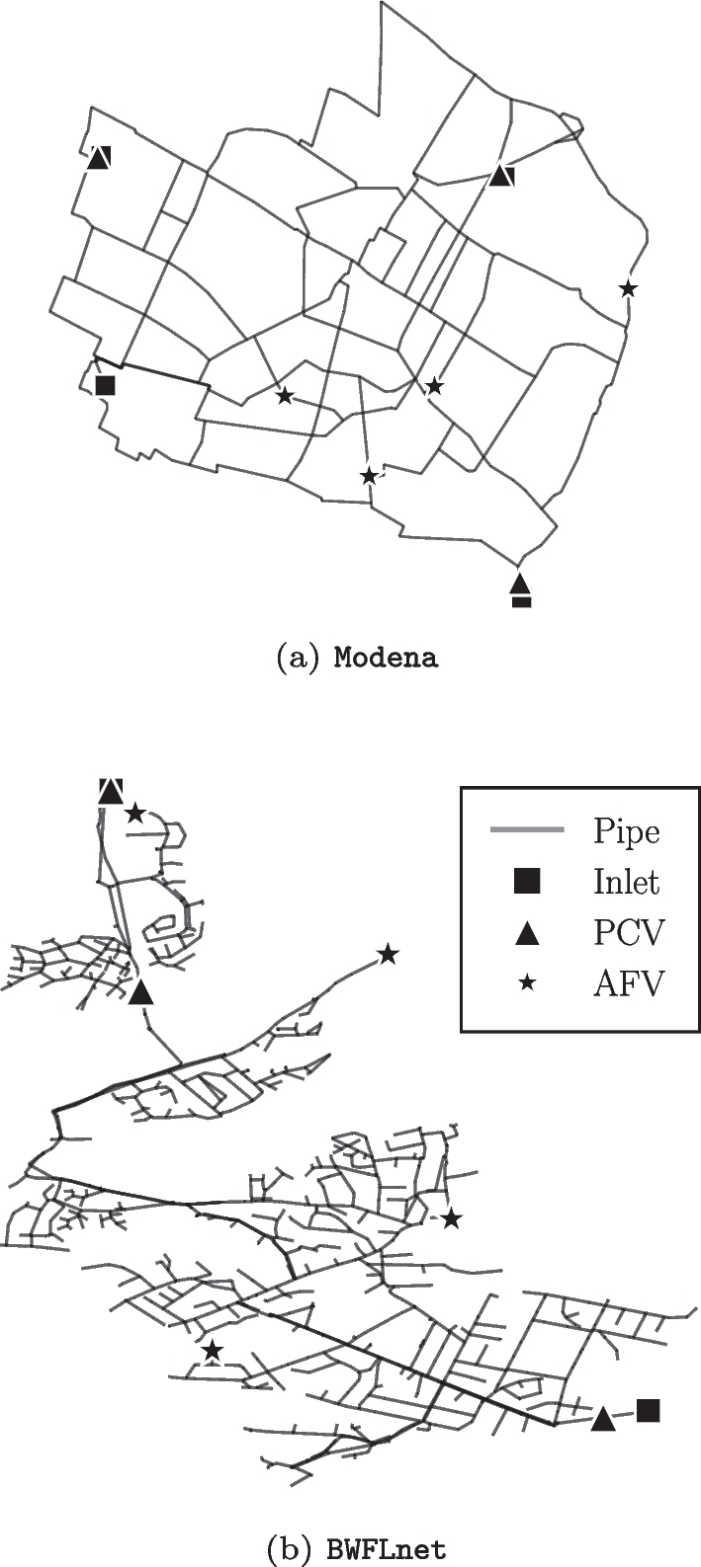
Case network layouts

We consider two case study networks: (i) Modena, a medium-sized benchmark model comprised of $n_0 = 4$ source nodes, ${n_n = 268}$ junction nodes, and ${n_p = 317}$ links (Bragalli et al. 2012); and (ii) Bristol Water Field Lab network (BWFLnet), a large-scale operational network model comprised of ${n_0 = 2}$ source nodes, ${n_n = 2745}$ junction nodes, and ${n_p = 2816}$ links (Wright et al. 2014). Both models feature $n_v = 3$ PCV and $n_f = 4$ AFV control actuators, with locations chosen *a priori* using the optimization method proposed in Jenks et al. (2023b). Figure 3 illustrates the networks’ topology and control valve locations.

Furthermore, we consider a 24-hour control horizon $\mathcal {T}$ to capture the diurnal periodicity of loading conditions (i.e. demands) in WDNs. For Modena, we use $n_t=24$ 1-hour time steps, which is a typical resolution for hydraulic modelling and existing control implementations. On the other hand, we use $n_t=96$ 15-minute time steps in BWFLnet to leverage higher resolution sensor data increasingly available in operational networks. Accordingly, control implementations in practice will need to handle increasingly larger optimization problem sizes. The sizes of the resulting optimization problems investigated in this work are summarized in Table 1.

**Table 1:** Problem sizes for case study networks

Network	# Cont. var.	# Lin. cons.	# Noncon. terms
Modena	14, 452	47, 932	8, 314
BWFLnet	537, 369	1, 862, 553	284, 800

The following parameters were chosen to formulate the time-coupled, nonconvex problem Eq. 12. We assume a 1-hour self-cleaning mode is activated during the morning peak demand period, as illustrated previously in Fig. 1. This is denoted by sets $\mathcal {T}_{\text {SCC}} = \{7, 8\}$ and $\mathcal {T}_{\text {SCC}} = \{38, \dots , 42\}$ for Modena and BWFLnet, respectively, where $\mathcal {T}_{\text {SCC}} \subset \mathcal {T}$. We applied a maximum flow rate $\alpha ^{\max } = 25 \, {\text {L}/\text {s}}$ at AFV actuators and a minimum regulatory pressure head of $h^{\min } = 15 \, {\text {m}}$ (UK regulations) at model nodes. Moreover, we considered three pressure range tolerances $\delta \in \{20, 15, 10\}$ (in meters) to define time-coupling constraint set $\bar{\mathcal {X}}$ in Eq. 10. These tolerances were selected with reference to the unconstrained case (i.e. $\bar{\mathcal {X}}=\mathbb {R}$) and the pressure range results reported in Rezaei et al. (2015) and Jara-Arriagada and Stoianov (2021).

### Implementation Details

All numerical experiments were written in Julia 1.9.1 (Bezanson et al. 2017) with optimization solvers accessed via JuMP 1.12.0 (Dunning et al. 2017). The code was compiled on a 2.50-GHz Intel(R) Core(TM) i9-11900H CPU with 8 cores and 32.0 GB of RAM running Ubuntu 20.04.5 LTS Linux distribution. Linear programs were solved using Gurobi 10.0.2 (Gurobi Optimization 2023), and nonlinear programs were solved using IPOPT 3.14.4 (Wächter and Biegler 2006) with the HSL MA57 linear solver (HSL 2021). Moreover, we used Julia’s Distributed.jl module to distribute the computations in Eqs. 16a and 21a across eight computing agents, each running their own instance of Julia. This approach was found to be faster than multithreading on a single process.

The two-level algorithm described in Algorithm 1 was implemented as follows. The dual variables $\lambda$ were bounded between $\underline{\lambda }=-10^{5}$ and $\overline{\lambda }=10^{5}$. We set $\gamma =1.25$ and $\omega =0.75$ for the penalty parameter $\beta ^{m+1}$ update in Eq. 24. To prevent scaling issues in IPOPT, we enforced a maximum penalty parameter of $\beta \le 10^5$. The inner level ADMM penalty parameter $\rho$ was initialized as $\rho ^m=2\beta ^m$ at each outer level iteration *m* (Sun and Sun 2023), except for inner level iteration $k=0$ where $\rho ^0 = 0$. We considered initial $\beta ^1$ values ranging from $10^{-3}$ to $10^2$ (see results in Section 5). Both the standard and two-level algorithms were initialized with feasible starting points $(q_t^1, h_t^1, u_t^1) \in \mathcal {X}_t$, for all $t \in \mathcal {T}$. We generated this solution from hydraulic states corresponding to the no control case (i.e. $u_t^1 = 0$, for all $t \in \mathcal {T}$). Moreover, the duplicated variables were initialized to $\bar{h}^1 = h^1$, satisfying the time-coupling constraints $\bar{\mathcal {X}}$ since pressure range tolerances $\delta$ were chosen on the basis of initial hydraulic conditions. For each outer level iteration *m*, ADMM was reinitialized using the previous (feasible) solution $(q^0, h^0, u^0):= (q^{m-1}, h^{m-1}, u^{m-1})$, and by setting $\bar{h}^0 = h^{m-1}$, $z^0=0$, and $y^0=-\lambda ^{m-1}$. We terminate the inner level ADMM when ${\Vert h^k - \bar{h}^k + z^k\Vert \le \sqrt{n_n n_t} \mathbin {/} (100 \cdot m)}$ or ${\Vert \rho ^m (z^{k-1} - z^k)\Vert \le 10^{-5}}$. The former criterion ensures that we satisfy primal feasibility, with a stopping tolerance that becomes increasingly strict with the outer iteration count *m*, while the latter checks the stability of slack variable $z^k$ to inform early ADMM termination. The standard ADMM and outer level ALM of the two-level method terminate when primal feasibility satisfies $\Vert h^m - \bar{h}^m\Vert \le \sqrt{n_n n_t} \cdot \epsilon _p$, where $\epsilon _p=10^{-2}$. This tolerance value was selected to align with the precision of pressure monitoring devices and the inherent uncertainties within hydraulic models.

## Results and Discussion

### Centralized Solver

We first discuss the performance of state-of-the-art nonlinear solver IPOPT (Wächter and Biegler 2006) for solving Eq. 12 in a centralized manner. The results for different pressure tolerances $\delta$ are summarized in Table 2. For the smaller Modena network, IPOPT successfully converged to a feasible solution in all problem instances with a maximum computational time on the order of 100 seconds. On the other hand, IPOPT failed to compute feasible solutions within the 1-hour time limit for the larger BWFLnet network when time-coupling constraints $\bar{\mathcal {X}}$ were imposed. This outcome was expected due to the substantial size of the resulting NLP problem formulated for BWFLnet with $n_t=96$ discrete time steps (see Table 1). Specifically, the dense nature of time-coupling constraints $\bar{\mathcal {X}}$ results in large memory requirements, making it challenging for IPOPT to handle efficiently. We also tested control periods with a smaller number of time steps for BWFLnet to better understand IPOPT’s limitations. Interestingly, there was a significant jump in computational time from 345 seconds to $> 3600$ seconds between problem instances with $n_t=24$ and $n_t=32$ time steps, respectively.

These results reveal that a centralized solution approach may not be practical for large-scale water networks. Consequently, distributed optimization becomes appealing to facilitate computationally efficient control strategies. Our experiments indicate that the computational bottleneck of centralized solvers is mainly due to the linear system solver’s memory usage within the interior-point solver. In this respect, first-order methods (e.g. sequential convex programming Wright et al. 2015) or matrix-free solvers (e.g. ALPAQA Pas et al. 2022) may offer viable alternatives to efficiently solve problem Eq. 12.

### Distributed Optimization

**Fig. 4:**
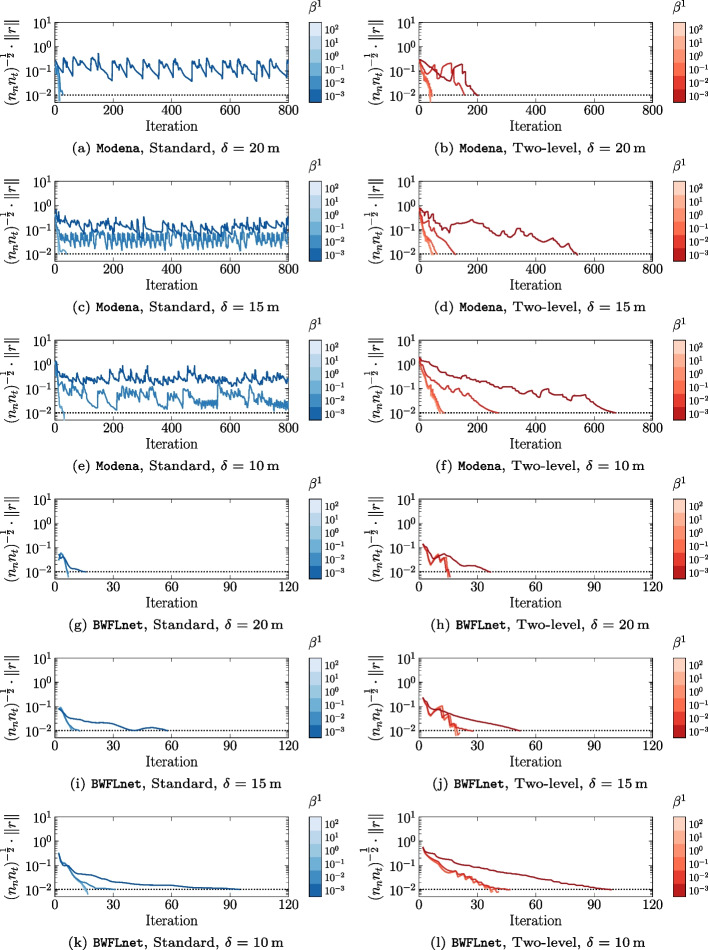
Convergence plots for all numerical experiments. Subplots correspond to experiments with different case network, distributed algorithm, and pressure tolerance $\delta$. Colour gradients delineate convergence behaviour for a range of initial penalty parameters $\beta ^1$ and the dotted black line represents convergence tolerance $\epsilon _p = 10^{-2}$

Figure 4 shows convergence plots for all numerical experiments using the standard and two-level ADMM algorithms. Each subplot corresponds to an experiment with different case network, distributed algorithm, and pressure range tolerance $\delta$.

Specifically, Fig. 4 illustrates the relationship between the average primal residual $(n_n n_t)^{-\frac{1}{2}} \cdot \Vert r\Vert$, where $r = h - \bar{h}$, and the number of ADMM iterations. Note that the cumulative number of inner ADMM iterations is used for the two-level algorithm. Within each subplot, initial penalty parameter $\beta ^1$ values are differentiated by a discrete colour bar and the dotted black line denotes the primal residual tolerance $\epsilon _p=10^{-2}$, indicating the algorithm’s successful termination.

The results show distinct convergence behaviour across the range of $\beta ^1$ values. For instance, larger $\beta ^1$ values have relatively fast and stable convergence. On the other hand, the number of iterations using small $\beta ^1$ values either increased significantly to meet the termination criterion or, in some cases, resulted in oscillatory behaviour and thus a failure to converge within the maximum number of iterations $k_{\max }=10^3$. The latter situation only appeared in experiments involving the Modena network and using the standard ADMM method with $\beta ^1 < 10^{-1}$. In these instances, the oscillations’ volatility and deviation from the termination criterion intensified as the pressure tolerance decreased. In contrast, the two-level algorithm consistently achieved convergence across all experiments. In exchange for algorithm robustness, however, the two-level algorithm exhibited a relatively slower convergence rate compared to the standard ADMM implementation. This behavior was more pronounced with smaller $\beta ^1$ values, especially in the Modena network. Modena’s network characteristics, including its highly looped structure and relatively equidistant PCV actuators, might be resulting in numerous local optima with similar objective values. In contrast, BWFLnet has a relatively branched structure, limiting the number of available flow paths for optimizing pressure or self-cleaning operations. Since water networks vary in topology and source conditions, tuning the penalty parameter for ADMM-based distributed algorithms is crucial for achieving good performance.

**Fig. 5:**
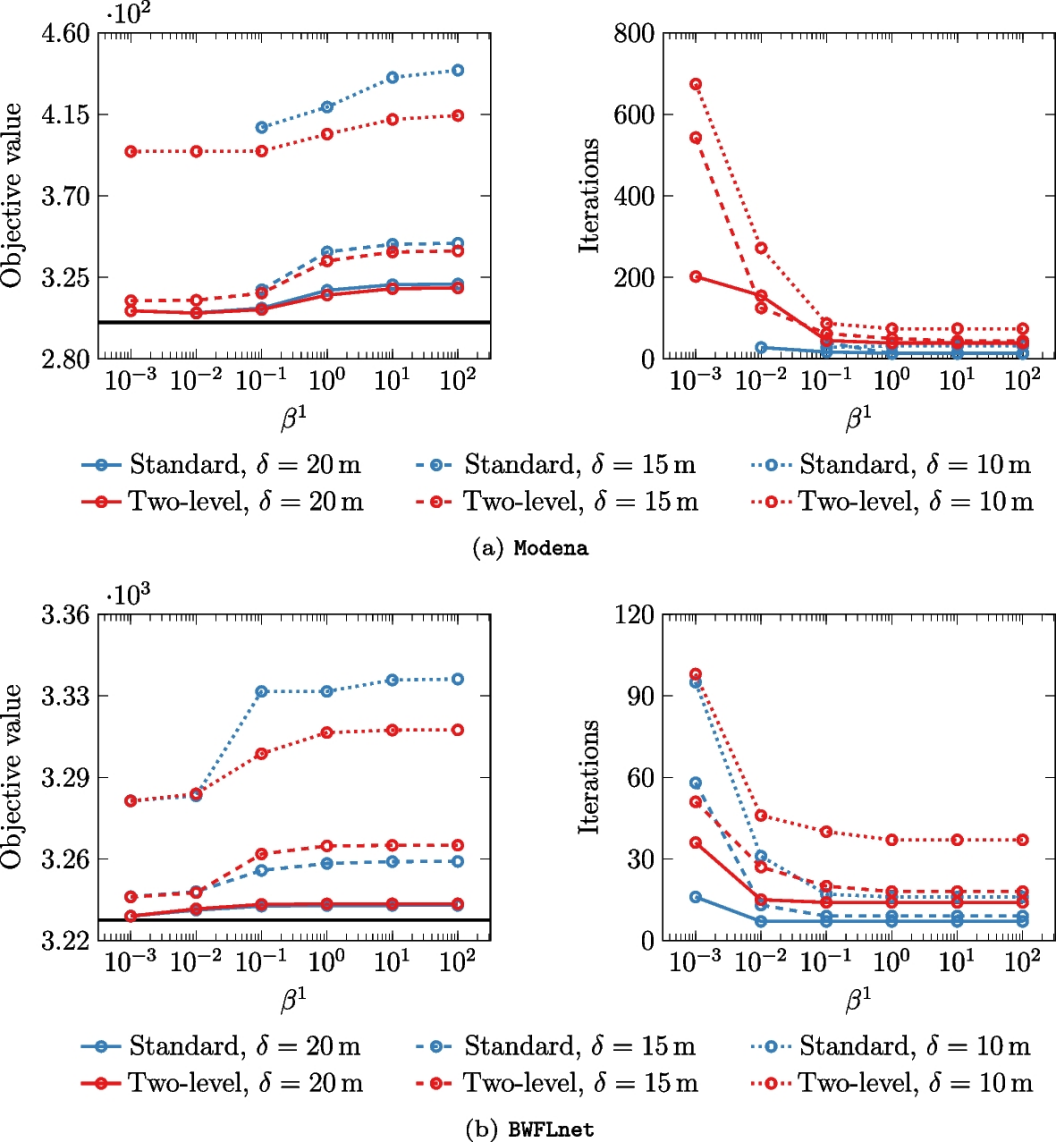
Distributed optimization results across a range of initial penalty parameters $\beta ^1$. Objective values and number of iterations are shown in the left and right plots, respectively. Pressure tolerances $\delta _i, \, \forall i \in \{20, 15, 10\}$ (in meters) are differentiated by line style. The solid black line is the unconstrained case (i.e. $\bar{\mathcal {X}}=\mathbb {R}$)

In Fig. 5, we explore the trade-off between objective value performance and iteration count for $\beta ^1$ values ranging from $10^{-3}$ to $10^2$.

Inflection points are observed at $\beta ^1=10^{-1}$ for Modena and $\beta ^1=10^{-2}$ for BWFLnet, marking the best trade-off between objective value and computational efficiency across the tested pressure tolerances. The results also show a strong agreement between the distributed algorithms when the standard ADMM method successfully converges, suggesting that cases with convergence issues can be discarded without compromising solution quality. From an operational perspective, Fig. 5 also highlights a notable reduction in objective value (and marginal decrease in iteration count) corresponding to higher pressure tolerances. The unconstrained case (solid black line) serves as a baseline to assess this improvement. Overall, tuning the penalty parameter is essential for optimizing performance across different networks and problem conditions.

The main advantage of the distributed optimization algorithms explored in this work is their fast convergence. It is important, however, to ensure that solution quality is not compromised to achieve this. In Table 2, we compare solutions computed by the centralized IPOPT solver with those obtained from the standard and two-level ADMM distributed algorithms. Note that the distributed algorithms apply the best $\beta ^1$ value derived from our penalty parameter tuning in Fig. 5 and the standard ADMM approach uses a fixed penalty parameter $\rho =2\beta ^1$.

**Table 2:** Comparison of centralized and distributed solution methods

Experiment			Centralized IPOPT		Distributed optimization				
Network	$\beta ^1$	$\delta$ [m]	Obj.	Time [s]	Method	Iter.	Obj.	$\delta _{\text {viol}}$ [m]	Time [s]
Modena	$10^{-1}$	$\infty$	298.3	28.4	Standard	1	300.1	0.00	13.6
					Two-level	1	300.1	0.00	25.5
		20	307.7	71.9	Standard	17	308.0	0.00	29.1
					Two-level	45	307.2	0.06	39.2
		15	312.9	98.5	Standard	40	318.2	0.32	29.8
					Two-level	62	316.3	0.32	45.9
		10	390.8	73.1	Standard	28	407.7	0.21	23.7
					Two-level	87	394.7	0.43	57.1
BWFLnet	$10^{-2}$	$\infty$	3228	1320	Standard	1	3229	0.00	78.0
					Two-level	1	3229	0.00	79.8
		20	−	−	Standard	7	3233	0.32	165
					Two-level	15	3234	0.92	425
		15	−	−	Standard	13	3241	0.85	291
					Two-level	27	3241	1.41	763
		10	−	−	Standard	31	3282	0.71	689
					Two-level	46	3283	0.58	1210

Tolerance $\delta =\infty$ corresponds to the unconstrained pressure range case (i.e. $\bar{\mathcal {X}}=\mathbb {R}$). Dashes denote experiments which failed to converge to a feasible solution within a 1-hour time limit

**Fig. 6:**
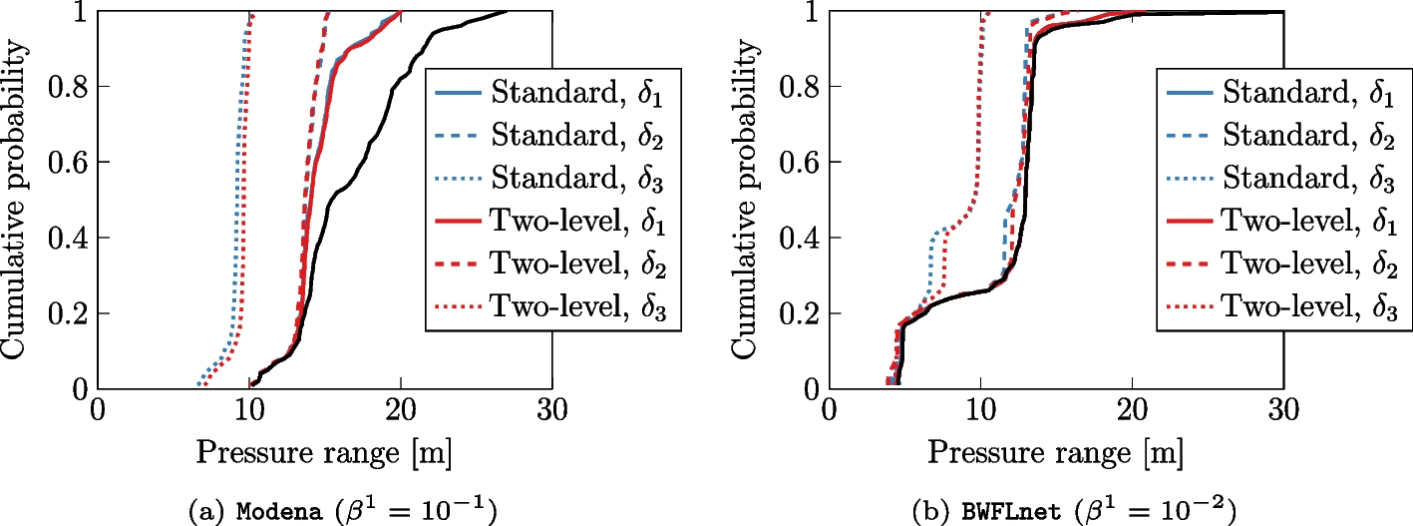
Cumulative distribution of temporal pressure range across network nodes. Distributed algorithm is depicted by plot colour and pressure tolerances $\delta _i, \, \forall i \in \{20, 15, 10\}$ (in meters) are differentiated by line style. The solid black line is the unconstrained case (i.e. $\bar{\mathcal {X}}=\mathbb {R}$)

In addition to objective value and computational time, Table 2 reports the cumulative number of inner ADMM iterations for the two-level algorithm, consistent with the presentation in Fig. 4. Additionally, we tabulate the maximum pressure constraint violations $\delta _{\text {viol}}$. These constraint violations were observed since both distributed algorithms employ a stopping criterion based on the Euclidean norm, which considers the overall distance of the solution from the feasible region rather than the maximum violation, leading to minor constraint violations. More specifically, less than $0.01\%$ of nodes failed to satisfy constraints $\bar{\mathcal {X}}$, with a maximum violation of $0.32 \, {\text {m}}$ and $1.41 \, {\text {m}}$ recorded for Modena and BWFLnet, respectively. It is worth noting that the two-level algorithm yielded slightly larger constraint violations in most experiments. In any case, these violations were considered acceptable due to their order of magnitude aligning with the inherent modeling uncertainties in water networks. Cumulative distribution plots in Fig. 6 provide a visualization of the temporal pressure range across the network for different tolerances. These plots highlight the limited impact of the maximum constraint violations within the broader context of the entire network.

Table 2 highlights the significant computational advantages of the distributed methods, particularly in generating feasible solutions for the large-scale BWFLnet experiments. Among these methods, the two-level algorithm showed only marginal improvements in solution quality over the standard ADMM approach. However, its computational times were typically two to three times longer than those of standard ADMM, especially in experiments involving BWFLnet with smaller pressure tolerances. This increase in computational time is due to the greater iteration complexity required by the two-level algorithm to ensure convergence for nonconvex problems. The two-level algorithm could offer an advantage in networks where frequent re-tuning of the penalty parameters might be necessary for convergence. Despite the longer computational times, the maximum reported time of 20 minutes across all experiments indicates that the two-level algorithm is a suitable (and robust) algorithm for near real-time (e.g. hourly) control in large-scale water networks.

## Conclusion

We presented a new control model for optimizing pressure and water quality operations in water distribution networks. The formulation imposed a set of time-coupling constraints to manage temporal pressure variations, which are exacerbated by the transition between pressure and water quality control objectives. We then investigated distributed optimization methods to solve the resulting time-coupled, nonconvex problem, as state-of-the-art nonlinear solvers struggled to find feasible solutions. In particular, we implemented two distributed optimization algorithms based on the alternating direction method of multipliers (ADMM): a standard ADMM scheme and a two-level variant that provided convergence guarantees for our nonconvex problem.

We evaluated the distributed algorithms using a benchmarking water network and a large-scale operational network in the UK. Our numerical experiments first demonstrated the sensitivity of convergence behaviour for different choices of the initial penalty parameter. This was highlighted by the standard ADMM method failing to converge for a number of experiments. Although the two-level algorithm proved to be more robust, finding feasible solutions to all numerical experiments, computational times increased significantly for small initial penalty parameters. With an appropriately tuned penalty parameter, however, both distributed algorithms yielded good quality solutions and computational times compatible with near real-time (e.g. hourly) control implementations. In future work, we aim to embed such efficient and robust solution methods within a model predictive control framework to optimize water network operations in near real-time.

## Data Availability

Data and code associated with this manuscript can be accessed at the following GitHub repository: https://github.com/bradleywjenks/wdn_control_admm.git.

## Appendix A Two-level Convergence Properties

The following presents theoretical convergence guarantees for solving problem Eq. 18 to an approximate stationary solution using the two-level algorithm.

### Remark 1

We show that Assumptions 1–3 provided in Sun and Sun (2023) hold for problem Eq. 18:

- **Assumption 1.** Problem Eq. 18 is feasible given that the chosen pressure range tolerance $\delta$ in Eq. 10 can be satisfied. We ensure $\delta$ is large enough by using the natural pressure variation from diurnal loading conditions as a baseline (i.e. no control).
- **Assumption 2.** The objective function *f*, which is a sum of linear and nonlinear functions $f_{\text {AZP}}$ and $f_{\text {SCC}}$, respectively, is continuously differentiable. Moreover, the sets $\mathcal {X}_t$ and $\bar{\mathcal {X}}$ are compact and defined by a finite number of constraints ${p_t = 3n_p+2n_n+2n_v+2n_f}$ and ${\bar{p} = n_tn_n + n_n}$, respectively. The condition that $\bar{\mathcal {X}}$ is convex is also met since it is comprised of linear constraints Eq. 11.
- **Assumption 3.** The first ADMM block update in Eq. 21a can find a stationary solution via efficient local optimization methods (Boyd and Vandenberghe 2004, Section 1.4.1.). In this work, we use state-of-the-art nonlinear solver IPOPT (Wächter and Biegler 2006), which applies an interior point line search filter method to compute local solutions to the nonlinear subproblems. Since we initialize ADMM with a feasible starting point (see line 5 in Algorithm 1), the updated iterate $(q^{k+1}, h^{k+1}, u^{k+1}, \bar{h}^k, z^k, y^k)$ is a stationary point of Eq. 21a with improved objective value. Thus,
  A1 $$\begin{aligned} \hat{L}^m(q^{k+1},h^{k+1},u^{k+1},\bar{h}^k,z^k,y^k) \le \hat{L}^m(q^k,h^k,u^k,\bar{h}^k,z^k,y^k) < \infty \end{aligned}$$
  holds at outer level iteration *m* for all $k \in \mathbb {Z}_{++}$.

Under Assumptions 1–3 in Remark 1, we can establish convergence guarantees for the two-level algorithm following (Sun and Sun 2023, Proposition 1 and Theorem 2). Specifically, if $\rho ^m=2\beta ^m$, then the sequence of solutions $\{(q^{m}, h^{m},u^{m},\bar{h}^m,z^{m},y^{m})\}_{m \in \mathbb {Z}_{++}}$ converge to a limit point $(q^{*}, h^{*},u^{*},\bar{h}^{*},z^{*},y^{*})$.

## Nomenclature

**Table 3:** List of key symbols and definitions

Symbol	Definition
$\mathcal {N}$	Set of model nodes
$\mathcal {P}$	Set of model links
$\mathcal {T}$	Set of time steps in control horizon
$n_n, n_0$	Number of junction and source nodes
$n_f$	Number of AFV nodes
$n_p$	Number of links
$n_v$	Number of PCV links
$n_t$	Number of discrete time steps
$A_{10}, A_{12}$	Link-source and -junction node incidence matrices
$A_{13}, A_{14}$	Matrix mappings of PCV and AFV locations
$d_t$	Nodal demands at time *t*
$h_{0t}$	Source hydraulic heads at time *t*
$q_t, h_t$	Flow and hydraulic head variables at time *t*
$u_t = (\eta _t, \alpha _t)$	PCV and AFV control variables at time *t*
$h^{\min }$	Minimum regulatory pressure head
$\alpha ^{\max }$	Maximum flow rate at AFV nodes
$f_t(\cdot )$	Control problem objective function at time *t*
$\zeta$	Node elevations
$\psi (\cdot )$	Sigmoid function in approximated SCC objective
$\mathcal {X}_t$	Set of hydraulic constraints at time *t*
$\bar{\mathcal {X}}$	Set of time-coupling constraints
$\delta$	Pressure range tolerance
$\bar{h}$	Duplicated hydraulic head variables for consensus constraint Eq. 13
*z*	Slack variables for ALR problem reformulation
$L(\cdot )$	Augmented Lagrangian function
*y*	Dual variables associated with constraints Eqs. 14b and 19b
$\lambda$	Dual variables associated with constraint Eq. 18e
$\rho$	Fixed ADMM penalty parameter
$\beta$	Adaptive ALM penalty parameter
$\gamma , \omega$	$\beta$ update parameters
$\epsilon$	Numerical convergence tolerance
